# Whole-body diffusion-weighted MRI for operability assessment in patients with colorectal cancer and peritoneal metastases

**DOI:** 10.1186/s40644-018-0187-z

**Published:** 2019-01-07

**Authors:** Raphaëla Carmen Dresen, Sofie De Vuysere, Frederik De Keyzer, Eric Van Cutsem, Hans Prenen, Ragna Vanslembrouck, Gert De Hertogh, Albert Wolthuis, André D’Hoore, Vincent Vandecaveye

**Affiliations:** 1 Department of Radiology, University Hospitals Leuven, KU Leuven, Herestraat 49, 3000 Leuven, Belgium; 2 Department of Digestive Oncology, University Hospitals Leuven, KU Leuven, Herestraat 49, 3000 Leuven, Belgium; 3 Department of Pathology, University Hospitals Leuven, KU Leuven, Herestraat 49, 3000 Leuven, Belgium; 4 Department of Abdominal Surgery, from the University Hospitals Leuven, KU Leuven, Herestraat 49, 3000 Leuven, Belgium

**Keywords:** WB-DWI/MRI, Colorectal peritoneal carcinomatosis, PCI, CT, FDG-PET/CT, CRS-HIPEC, Operability assessment, Metastases

## Abstract

**Background:**

Correct staging of patients with colorectal cancer is of utmost importance for the prediction of operability. Although computed tomography (CT) has a good overall performance, estimation of peritoneal cancer spread is a known weakness, a problem that cannot always be overcome by Fluorine-18 fluorodeoxyglucose positron emission tomography/computed tomography (^18^F-FDG-PET/CT); especially in infiltrative and miliary disease spread. Due to its high spatial and contrast resolution magnetic resonance imaging (MRI) with diffusion-weighted imaging (DWI) might have a better performance. Our aim was to evaluate the added value of whole-body diffusion-weighted MRI (WB-DWI/MRI) to CT for prediction of peritoneal cancer spread and operability assessment in colorectal cancer patients with clinically suspected peritoneal carcinomatosis (PC).

**Methods:**

This institutional review board approved retrospective study included sixty colorectal cancer patients who underwent WB-DWI/MRI in addition to CT for clinically suspected peritoneal metastases. WB-DWI/MRI and CT were assessed for detecting PC following the peritoneal cancer index (PCI), determination of PCI-score categorized as PC < 12, PCI = 12–15 and PCI > 15, detection of nodal and distant metastases and estimation of overall operability. Histopathology after surgery and biopsy and/or 6 months follow-up were used as reference standard.

**Results:**

For detection of PC, CT had 43.2% sensitivity, 95.6% specificity, 84.5% positive predictive value (PPV) and 75.2% negative predictive value (NPV). WB-DWI/MRI had 97.8% sensitivity, 93.2% specificity, 88.9% PPV and 98.7% NPV. WB-DWI/MRI enabled better detection of inoperable distant metastases (all 12 patients) than CT (2/12 patients) and significantly improved prediction of PCI category [WB-DWI/MRI PCI < 12: 37/39 patients (94.9%); PCI = 12–15: 4/4 patients (100%); PCI > 15: 16/17 patients (94.1%) versus CT PCI < 12: 38/39 patients (97.4%); PCI = 12–15: 0/4 patients (0%); PCI > 15: 2/17 patients (11.8%); *p* < 0.0001)]. WB-DWI/MRI improved prediction of inoperability over CT with 90.6% sensitivity compared to 25% (*p* < 0.0001).

**Conclusions:**

WB-DWI/MRI significantly outperformed CT for estimation of spread of PC, overall staging and prediction of operability. Pending validation in larger prospective trials, WB-DWI/MRI could be used to guide surgical planning and minimize unnecessary exploratory laparotomies.

## Background

Around 10% of patients with primary and 25% with recurrent colorectal cancer present with peritoneal carcinomatosis (PC) [1]. These patients have a poor prognosis when treated with systemic chemotherapy alone [2, 3]. Aside from optimization of chemotherapy regimens, new aggressive invasive therapeutic strategies have been introduced. Cytoreductive surgery (CRS) followed by hyperthermic intraperitoneal chemotherapy (HIPEC) aims at removing all visible and invisible peritoneal metastases and can achieve an encouraging median survival of 5 years [4, 5]. Strict patient selection is mandatory to balance clinical benefit with treatment related morbidity and mortality [6]. Therefore, accurate staging of tumour burden is essential in these patients.

Currently, PC assessment is mainly performed during exploration at CRS. However, with reported non-therapeutic open-close procedures of 20–40% [7, 8], accurate preoperative staging is needed. Although laparoscopy may be considered when widespread peritoneal disease is suspected, it is not routinely recommended [9]. The procedure is invasive and can only inform on the accessible parts of the peritoneal cavity. Disease spread in regions difficult to reach by laparoscopy and regions outside the peritoneal cavity (necessary to assess for overall operability prediction) remains undetected with this procedure. Computed tomography (CT) is most frequently used for staging colorectal cancer patients, allowing rapid simultaneous evaluation of disease spread in the chest and abdomen. Although CT has a good accuracy for detecting liver and lung metastases, estimation of PC is suboptimal, due to its limited soft tissue contrast resolution [10, 11]. ^18^F-Fluoro-deoxyglucose positron emission tomography (FDG-PET)/CT can only partially overcome this problem as its lower spatial resolution limits sensitivity, especially in small volume disease [12, 13]. Therefore, both techniques have limitations for treatment planning [14].

Diffusion-weighted magnetic resonance imaging (DWI/MRI) has the potential to solve this problem. It is a functional imaging technique that generates image contrast based on differences in water proton mobility within a voxel of tissue. In tumour tissue this mobility is limited because of the inherent high cellularity, leading to lower diffusion coefficients and higher signal intensities on high b values as opposed to the normal surrounding tissue where the water protons can freely move and generate high diffusion coefficients. The large microstructural differences between tumour and normal tissue allow tumour detection at millimetre level. DWI significantly improves peritoneal tumour depiction, particularly for mesenterial/serosal disease [15]. It can be used as a whole body (WB) imaging technique, allowing assessment of primary tumour and metastases in one clinically time-efficient examination [16, 17].

Our aim was to evaluate the added value of WB-DWI/MRI to CT for detection of PC, overall staging and prediction of operability in patients with suspected PC from colorectal cancer.

## Methods

### Patients

This retrospective was approved by the institutional review board. Informed consent was waived. Between April 2011 and March 2016, 73 consecutive patients with primary or recurrent colorectal cancer with a clinical suspicion of peritoneal metastases underwent a WB-DWI/MRI for the evaluation of operability for HIPEC surgery in addition to conventional staging by CT. Thirteen patients received chemotherapy between the CT and the WB-DWI/MRI and were excluded from analysis.

### Computed tomography

Breath-hold CT scans (Sensation 16 or Sensation 64, Definition Flash, Siemens Medical Systems, Erlangen, Germany), were acquired with intravenous (70s after 120 ml iodinated contrast injection, Visipaque, GE Healthcare) and oral (30 ml iodinated contrast agent (Telebrix Gastro, Guerbet), 300 mg/ml, in 900 ml water) contrast in the transverse plane. The following parameters were used: pitch 1.2, rotation speed 0.5 s, 5 mm slice thickness, 1 mm slice gap and 0.6 mm collimation. A reference current of 110 mAs (thorax) and 200 mAs (abdomen) was used using automated Care Dose software. The images were reconstructed coronally (3 mm).

### WB-DWI/MRI

All WB-DWI/MRI examinations were performed on a 3 Tesla scanner (Ingenia, Philips Healthcare, Best, The Netherlands) with parallel radiofrequency transmission and phased-array surface coils. Free-breathing transverse diffusion-weighted images were acquired in four imaging stations (head/neck, thorax, abdomen and pelvis) at b = 0 and b = 1000 s/mm^2^. Coronal free-breathing single shot Turbo spin-echo T2-weighted images and breath-hold gadolinium-enhanced (Dotarem®, Guerbet, Roissy, France) 3D T1-weighted spoiled gradient-echo sequences were acquired for thorax, abdomen and pelvis. Patients drank one litre of pineapple juice two hours before the WB-DWI/MRI and received antispasmodic medication (butylhyoscine, 20 mg IV) to minimize high signal intensities on the diffusion-weighted images from bowel content and bowel movement. The details of the imaging protocol are displayed in Table 1.

**Table 1:** Detailed sequence parameters of WB-DWI/MRI (whole body diffusion-weighted magnetic resonance imaging)

	DWI			T2 SSTSE	Contrast-enhanced 3D T1 gradient-echo		
	Transverse	Coronal	Sagittal	Coronal	Transverse	Coronal	Transverse
Image stations head to mid-thigh	4	MPR	MPR	3	abdominopelvic (2)	abdominopelvic (2)	Chest (1)
Respiration	Free breathing			respiratory	15 s breath-hold		
Fat suppression	STIR (TI = 250 ms)			none	SPAIR (mDIXON)	SPAIR (mDIXON)	SPAIR (eTHRIVE)
b-values (s/mm^2^)	0–1000			none	none	none	none
Parallel imaging factor	2.5			4	2	2	2
Repetition time (TR) (ms)	8454			3000	3.6	3.6	3.2
Echo time (TE) (ms)	67			87	1.25–2.20	1.25–2.20	1.5
Slice thickness (mm)	5	5	5	6	2.5	2.5	2.5
Slice number	50/station			35/station	90	133	148
Intersection gap (mm)	0.1			0.6	0	0	0
Field of view (FOW) (mm)	420 × 329			375 × 447	375 × 304	400 × 352	375 × 304
Acquired voxel size (mm)	4.57 × 4.71			1 × 1	1.49 × 1.5	1.49 × 1.5	1.49 × 1.5
Reconstructed voxel size (mm)	2.19 × 2.16			0.93 × 0.93	0.71 × 0.71	0.71 × 0.71	0.98 × 0.97
Number of signal averages (NSA)	1			1	1	1	1

*DWI* diffusion-weighted imaging, *mDIXON* multi-echo 2-point Dixon, *eTHRIVE* T1-weighted high-resolution isotropic volume examination, *SSTSE* single-shot turbo spin-echo imaging, *STIR* short T1 inversion recovery, *SPAIR* spectrally adiabatic inversion recovery

### (Pre-)operative evaluation

**Fig. 1:**
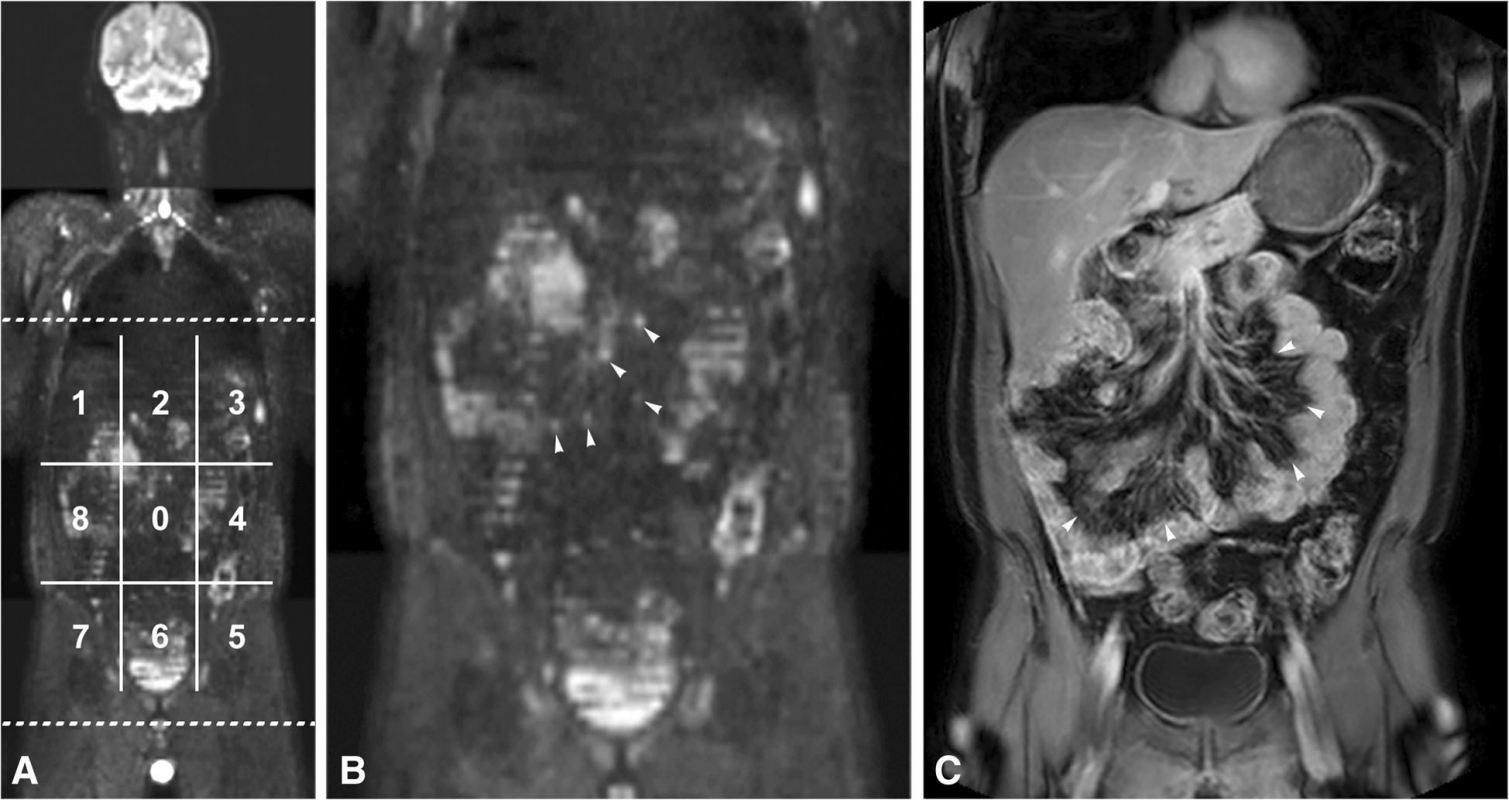
WB-DWI/MRI in a 56 year old male patient. On image A 9 of the 13 regions of the peritoneal cancer index (PCI) are displayed (regions 0–8). Region 9 is the proximal jejunum, region 10 the distal jejunum, region 11 the proximal ileum and region 12 the distal ileum (not displayed on the image). This patient was diagnosed with a caecal adenocarcinoma with extensive peritoneal metastases, for which he received chemotherapy during 5 months. Afterwards, a WB-DWI/MRI was performed to evaluate operability. On the coronal b1000 DWI (**a**), with a zoomed-in image (**b**) and the correlating coronal post-contrast T1-weighted image (**c**), good therapy response was seen with residual disease on the small bowel wall (arrowheads in **c**) with accompanying limited nodules with diffusion restriction in the small bowel mesentery (arrowheads in **b**). The patient was given the benefit of the doubt and underwent surgery. Surgery, however, revealed diffuse serosal metastases in the small bowel, limiting curative resection. Instead, optimal palliative care was provided. This patient was scored as a false negative interpretation of the WB-DWI/MRI

The peritoneal cancer index (PCI) as proposed by Jacquet and Sugarbaker [18] was used as scoring system on CT, on WB-DWI/MRI and during surgery. With this scoring system the peritoneal cavity is divided into 13 areas (Fig. 1), giving each area a score from 0 to 3 (0 = no peritoneal metastases, 1 = metastases< 5 mm, 2 = metastases of 5 mm-5 cm, 3 = confluent metastases/> 5 cm), with a maximum score of 39. The PCI was categorized as followed: PCI < 12 (operable peritoneal), PCI = 12–15 (operability decided by the multidisciplinary team) and PCI > 15. Patients were deemed primarily inoperable if the PCI was > 15 and/or the following areas were involved: infiltration of the mesenteric root, invasion of the gastrohepatic ligament, extensive involvement of the small bowel (estimated residual length < 120 cm after resection), inaccessible lymph node involvement or inoperable distant metastases.

### Evaluation of imaging

CT and WB-DWI/MRI were reviewed separately, by two abdominal radiologists (18 and 15 years of experience respectively) for identification of possible lymphadenopathies, peritoneal and/or distant metastases. Peritoneal disease spread was recorded at CT and MRI as mentioned above. Both readers were blinded to all information regarding each other’s imaging results, clinical, laboratory, surgical and pathological findings.

At CT, peritoneal metastases were recorded in the presence of nodular, confluent or infiltrative contrast-enhancing lesions involving the peritoneum, omentum or mesentery. Serosal metastases were recorded in case of contrast-enhancing lesions at the bowel wall, bowel wall thickening or increased contrast-enhancement. Lymph nodes were characterized based on the short axis diameter (with 1 cm as cut-off level) and nodal irregularity or abnormal contrast-enhancement indicating presence of necrosis or cystic change. Distant metastases were identified based on established CT-graphic criteria for lung and liver lesions.

WB-DWI/MRI combined the information of b1000 diffusion-weighted images and anatomical sequences. Peritoneal metastases were recorded when there was nodular, infiltrative or confluent b1000 hyperintensity and/or contrast enhancement over the peritoneal surfaces, omentum or mesentery. Involvement of the bowel serosa was recorded in case of b1000 and/or contrast-enhancing bowel wall masses, nodular or infiltrative thickening of the bowel wall. Lymph nodes were qualitatively assessed based on shape and b1000 signal intensity; lymph nodes showing (heterogeneous) higher b1000 signal intensity than the surrounding lymph nodes and visible on the anatomical images as round instead of oval were considered malignant. Distant metastases were identified based on increased b1000 signal not attributable to physiologically impeded diffusion or T2 shine through.

### CRS and HIPEC

Surgery started with exploration of the abdominal cavity and PCI was estimated with or without taking biopsies. If the patient was deemed operable a macroscopically complete cytoreductive surgery was performed, followed by the HIPEC procedure. When the hyperthermic perfusion reached a steady state of 41–42 °C the intraperitoneal drug was added to the perfusion (oxaliplatin at a dose of 460 mg/m^2^ during 30 min). One hour before starting the HIPEC procedure, Folinic Acid 20 mg/m^2^ and 5-fluorouracil 400 mg/m^2^ (in 250 ml saline) were administered intravenously to enhance the effect of oxaliplatin. When necessary, anastomoses were performed after the HIPEC procedure was finalized.

### Reference standard

Exploration during laparotomy/laparoscopy with histopathology was the primary reference standard. In case of suspected distant or retroperitoneal nodal metastases critical towards salvage surgery, image-guided biopsy was performed. If surgical/histopathological correlation was impossible, imaging follow-up for at least 6 months or correlative imaging with FDG-PET/CT was used.

### Statistical analysis

Sensitivity, specificity, positive predictive value (PPV) and negative predictive value (NPV) of CT and WB-DWI/MRI for prediction of disease occurrence in the different regions of the abdominal cavity and for prediction of inoperability were calculated. Correlations between CT or WB-DWI/MRI with the reference standard were examined using Chi-square analysis. Comparison between CT and WB-DWI/MRI was performed with the McNemar test. All statistical analyses were performed with SPSS® for Windows Release 24.0 (SPSS Inc., Chicago, IL, USA). A *P* value of < 0.05 was assumed to indicate statistical significance.

## Results

### Patient, tumour and treatment characteristics (Table 2)

Of the 60 patients, 32 patients were deemed inoperable, because of high PCI (*n* = 16), peritoneal location (*n* = 4), inoperable distant metastases (*n* = 8) and the combination of high PCI and distant metastases (*n* = 4). Nine of these 32 patients were already clearly inoperable at CT and WB-DWI/MRI and did not undergo surgery. The other 23 patients were found to be inoperable during surgical exploration. Distant metastases were found in 16 patients: liver (*n* = 10), lungs (*n* = 2), mediastinum (*n* = 1), abdominal wall (*n* = 3). Of these, 10 were confirmed by histopathology during surgical exploration and 6 during follow-up imaging.

**Table 2:** Patient, tumour and treatment related characteristics

*N* = 60	
Mean age, yrs. (range)	56 (25–81)
Gender	
Male	26
Female	34
Colorectal cancer	
Primary	23
Before chemotherapy	12
After chemotherapy	11
Recurrent	37
Before chemotherapy	28
After chemotherapy	9
Reference standard PCI	
< 12	39
12–15	4
> 15	17
Operable	28
Inoperable	32

*PCI* peritoneal cancer index according to Jacquet and Sugarbaker

### Operability assessment

#### PCI

**Fig. 2:**
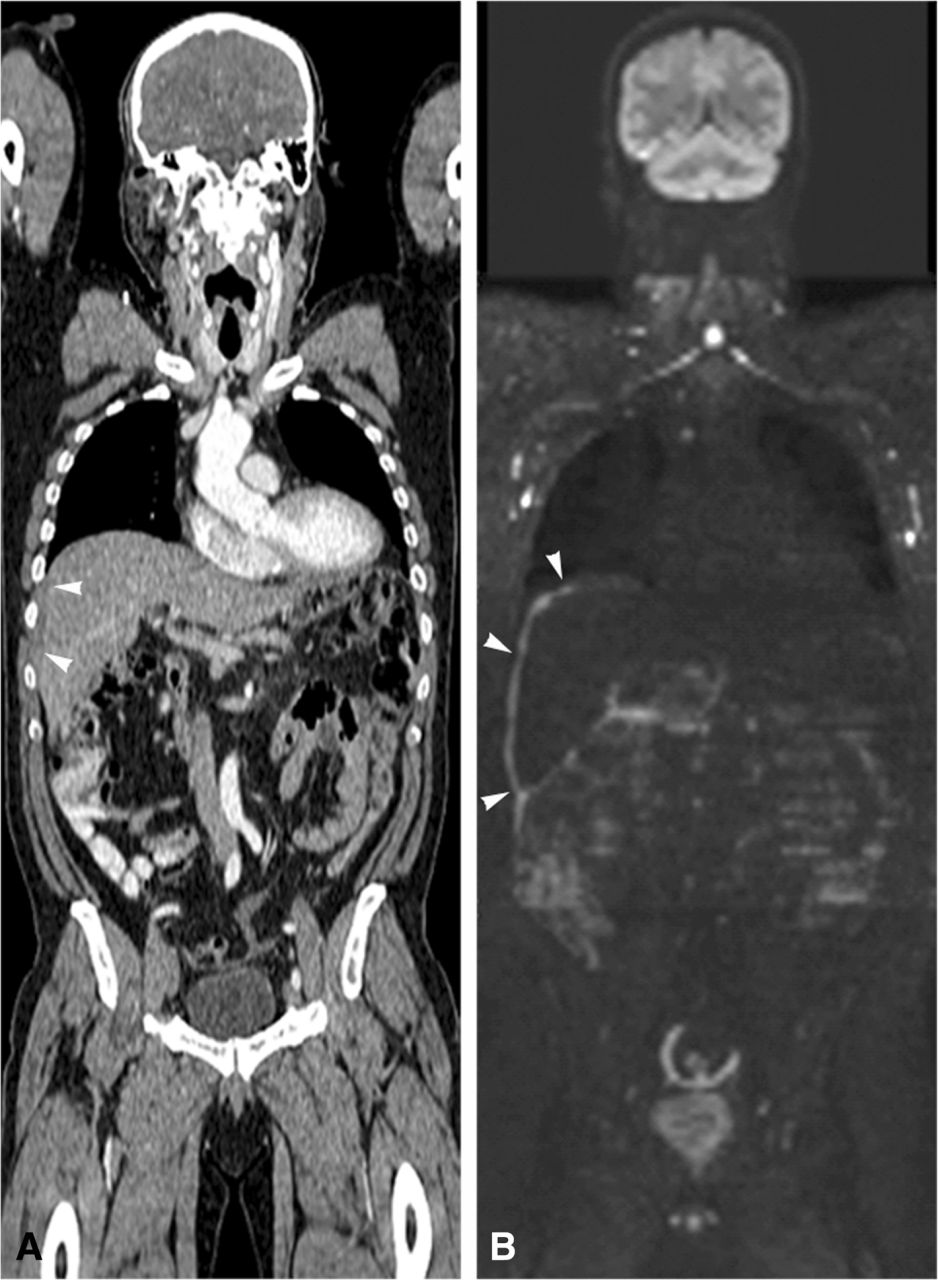
Coronal CT (**a**) and WB-DWI/MRI b1000 (**b**) image of a 73 year old male patient with a primary tumour in the descending colon and synchronous peritoneal carcinomatosis. CT images estimated the PCI to be 7 (partially shown with the arrowheads in A as a hypodense thickening on the liver surface) and WB-DWI/MRI estimated a PCI of 19 (partially shown with the arrowheads in B as diffuse confluent metastases at the liver surface). The high PCI of 19 was confirmed during explorative laparotomy. The patient was deemed inoperable and received palliative chemotherapy

**Fig. 3:**
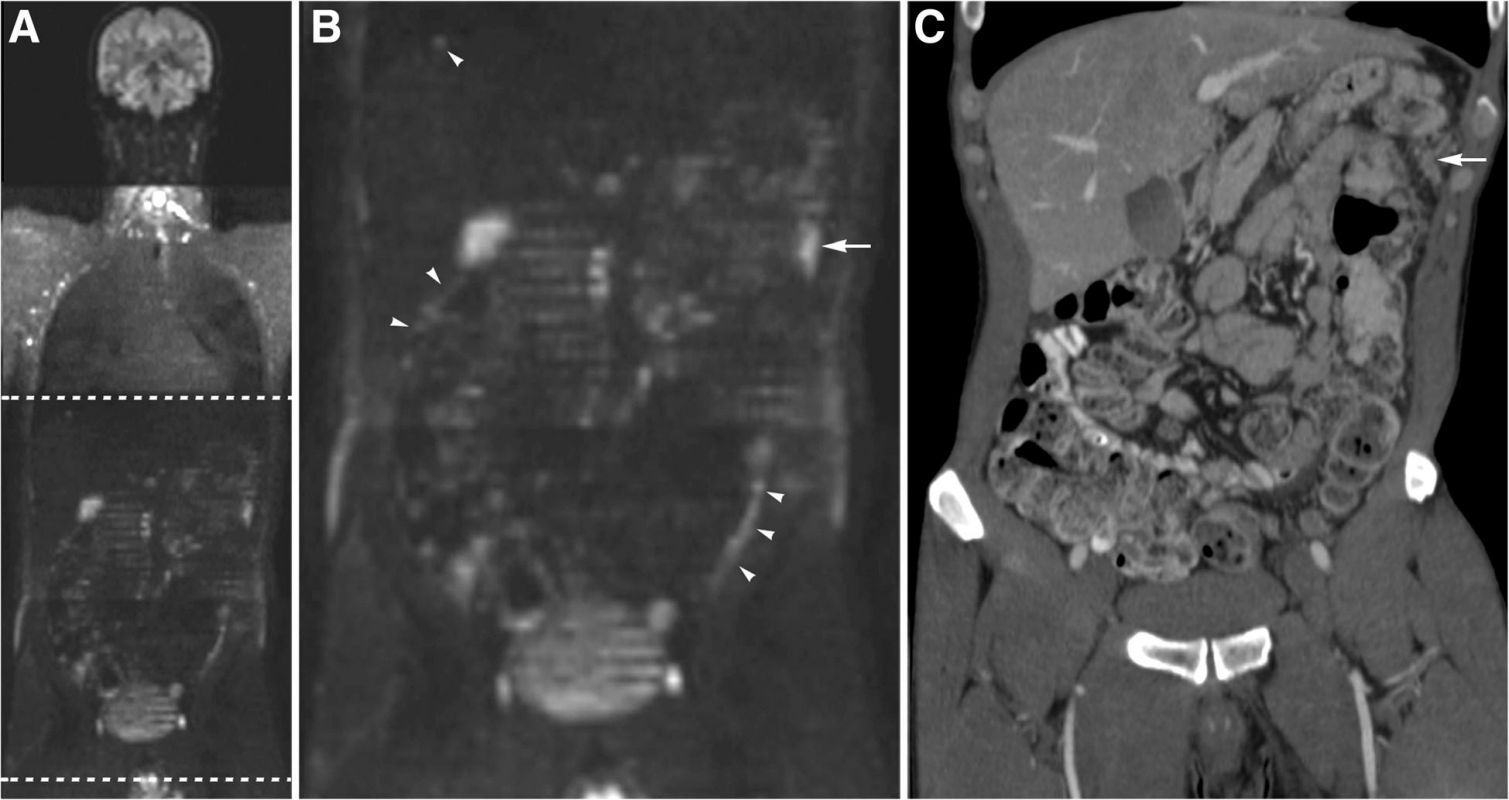
A 51 year old male patient with cancer at the rectosigmoid junction and clinical suspicion of peritoneal metastases underwent a WB/DWI-MRI (**a**/**b**) in addition to a CT scan (**c**) to evaluate operability. WB/DWI-MRI showed extensive peritoneal cancer spread, partially shown by the white arrowheads in the zoomed-in image (**b**) of the coronal b1000 DWI (**a**), with an estimated PCI of 26. The tumour deposit at the splenic flexure of the colon (arrow in **b**) was also recognized on CT, shown by the white arrow on the coronal CT image (**c**), but the other lesions were not seen on CT. Disease load was clearly underestimated with an estimated PCI of 8. At explorative laparotomy extensive peritoneal disease was confirmed (PCI 33) and the patient received optimal palliative care

The PCI category estimated on imaging had a significant correlation with the PCI category according to the reference standard (CT: *P* = 0.01, WB-DWI/MRI: *P* < 0.0001). WB-DWI/MRI was significantly better than CT for the prediction of PCI category (57/60 versus 30/60, *P* = 0.0002), see examples in Figs. 2 and 3. For a PCI of < 12 WB-DWI/MRI was correct in 94.9% of the patients (37/39), CT in 97.4% (38/39). For a PCI of 12–15 WB-DWI/MRI was 100% correct (4/4), CT 0% (0/4). WB-DWI/MRI correctly staged 16/17 patients with a PCI > 15 (94.1%), CT 2/17 (11.8%).

#### Location of peritoneal disease

To identify metastases in the 13 different regions defined by the PCI CT had an overall sensitivity of 43.2%, specificity 95.6%, PPV 84.5%, NPV 75.2%. For WB-DWI/MRI these values were 97.8, 93.2, 88.9 and 98.7%, respectively. The values per region for CT and WB-DWI/MRI are displayed in detail in Table 3.

**Table 3:** Accuracy of WB-DWI/MRI and CT for prediction of disease prevalence in the 13 peritoneal regions of the abdominal cavity

Region	WB-DWI/MRI				CT			
	Sens	Spec	PPV	NPV	Sens	Spec	PPV	NPV
0	92.9 (26/28)	90.6 (29/32)	89.7 (26/29)	93.5 (29/31)	57.1 (16/28)	90.6 (29/32)	84.1 (16/19)	70.7 (29/41)
	76.5–99.1	75.0–98.0	74.6–96.2	79.2–98.2	37.1–75.5	75.0–98.0	63.4–94.3	60.8–79.0
1	90.5 (19/21)	92.3 (36/39)	86.4 (19/22)	94.7 (36/38)	33.3 (7/21)	100 (39/39)	100 (7/7)	73.6 (39/53)
	69.6–98.8	79.1–98.4	67.9–95.0	82.8–98.5	14.6–57.0	91.0–100		67.3–79.0
2	100 (11/11)	95.9 (47/49)	84.6 (11/13)	100 (47/47)	54.5 (6/11)	98 (48/49)	85.7 (6/7)	90.6 (48/53)
	71.5–100	86.0–99.5	58.6–95.5		23.4–83.3	89.2–100	44.5–97.8	83.4–94.8
3	100 (16/16)	95.5 (42/44)	88.9 (16/18)	100 (42/42)	43.8 (7/16)	95.5 (42/44)	77.8 (7/9)	82.4 (42/51)
	79.4–100	84.5–99.4	67.4–96.9		19.8–70.1	84.5–99.4	44.8–93.8	75.1–87.8
4	100 (21/21)	94.9 (37/39)	91.3 (21/23)	100 (37/37)	38.1 (8/21)	94.9 (37/39)	80 (8/10)	74 (37/50)
	83.9–100	82.7–99.4	73.1–97.6		18.1–61.6	82.7–99.4	48.3–94.5	66.9–80.1
5	100 (24/24)	94.4 (34/36)	92.3 (24/26)	100 (34/34)	25 (6/24)	97.2 (35/36)	85.7 (6/7)	66 (35/53)
	85.8–100	81.3–99.3	75.7–97.9		9.8–46.7	85.5–99.9	43.5–97.9	60.5–71.2
6	97.3 (36/37)	95.7 (22/23)	97.3 (36/37)	95.7 (22/23)	56.8 (21/37)	91.3 (21/23)	91.3 (21/23)	56.8 (21/37)
	85.8–99.9	78.1–99.9	84.1–99.6	76.1–99.4	39.5–72.9	72.0–98.9	73.1–97.6	47.1–66.0
7	100 (20/20)	87.5 (35/40)	80 (20/25)	100 (35/35)	35 (7/20)	92.5 (37/40)	70 (7/10)	74 (37/50)
	83.2–100	73.2–95.8	63.8–90.1		15.4–59.2	79.6–98.4	40.3–89.0	67.1–79.9
8	96.2 (25/26)	94.1 (32/34)	92.6 (25/27)	97 (32/33)	65.4 (17/26)	91.2 (31/34)	85 (17/20)	77.5 (31/40)
	80.4–99.9	80.3–99.3	76.5–98.0	82.4–99.6	44.3–82.8	76.3–98.1	65.0–94.5	66.8–85.5
9	100 (14/14)	95.7 (44/46)	87.5 (14/16)	100 (44/44)	28.6 (4/14)	100 (46/46)	100 (4/4)	82.1 (46/56)
	76.8–100	85.2–99.5	64.4–96.5		8.4–58.1	92.3–100		76.8–86.5
10	100 (17/17)	93 (40/43)	85 (17/20)	100 (40/40)	17.7 (3/17)	97.7 (42/43)	75 (3/4)	75 (42/56)
	80.5–100	80.9–98.5	65.6–94.4		3.8–43.4	87.7–99.9	25.1–96.4	70.6–79.0
11	100 (18/18)	90.5 (38/42)	81.8 (18/22)	100 (38/38)	33.3 (6/18)	95.2 (40/42)	75 (6/8)	76.9 (40/52)
	81.5–100	77.4–97.3	63.9–92.0		13.3–59.0	83.8–99.4	40.1–93.1	70.5–82.3
12	100 (29/29)	90.3 (28/31)	90.6 (29/32)	100 (28/28)	51.7 (15/29)	96.8 (30/31)	93.8 (15/16)	68.2 (30/44)
	88.1–100	74.3–98.0	76.7–96.6		32.5–70.6	83.3–99.9	67.9–99.1	59.4–75.9

*WB-DWI/MRI* magnetic resonance imaging, *CT* computed tomography, *Sens* sensitivity, *Spec* specificity, *PPV* positive predictive value, *NPV* negative predictive value

Numbers are displayed as percentages with confidence intervals, absolute numbers between brackets. Region; region of the abdominal cavity as defined by Jaquet and Sugarbaker^18^

#### Lymphadenopathies and distant metastases

The only 2 patients with retroperitoneal lymphadenopathies were correctly identified by CT and WB-DWI/MRI.

**Fig. 4:**
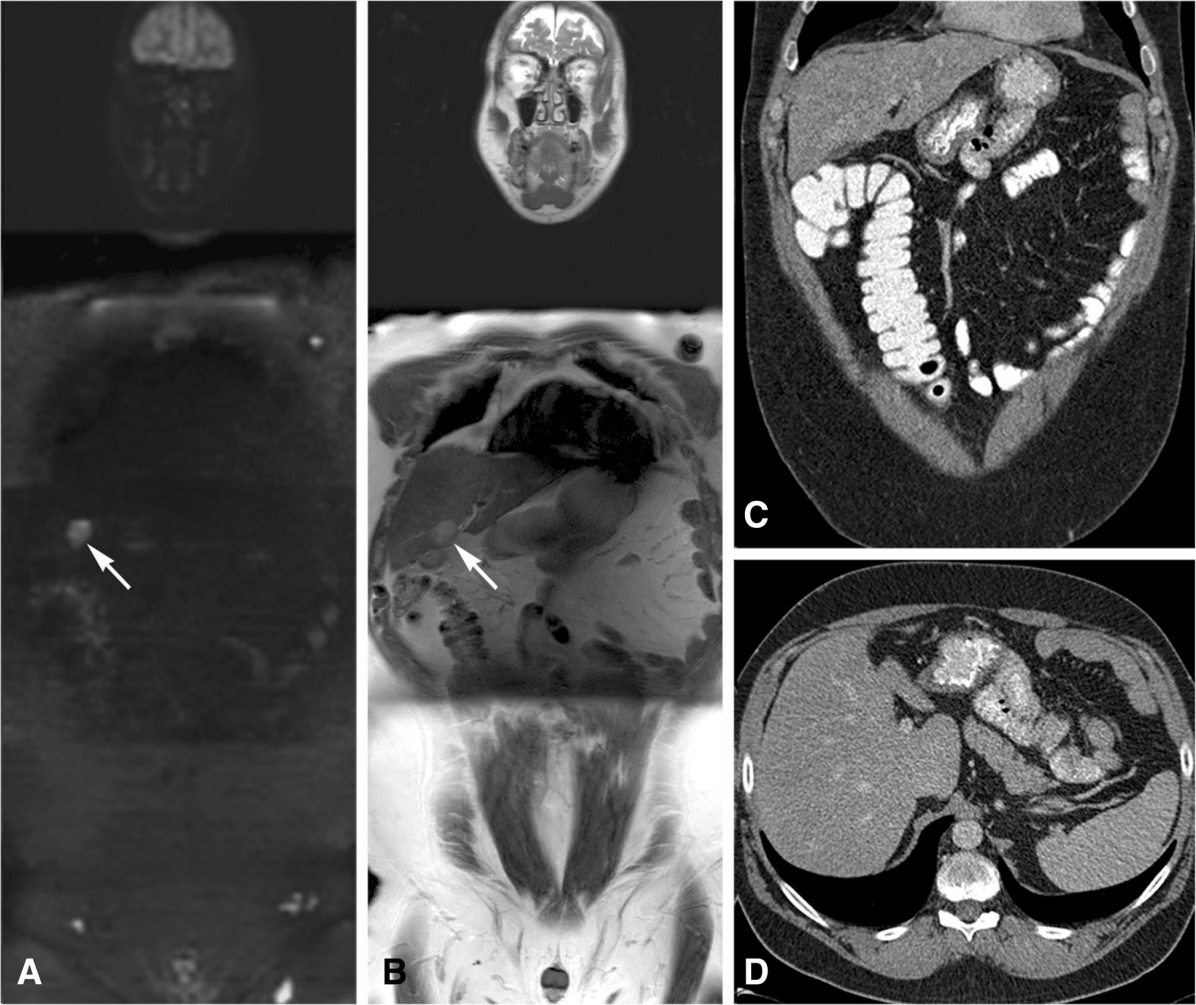
A 49 year old male patient with a history of a sigmoid resection because of adenocarcinoma was diagnosed with tumour recurrence. He had a WB-DWI/MRI for operability assessment. Apart from limited peritoneal disease (not shown on the images) a liver metastasis was found in liver segment 4B, shown with the arrows on the coronal b1000 DWI (**a**) and the coronal T2-weighted image (**b**). This liver metastasis was not recognised on the axial (**c**) and coronal (**d**) CT images. The patient could undergo a curative debulking with RFA of the liver metastasis

CT failed to identify inoperable distant metastases in 2 patients with low PCI (> 3 liver metastases: *n* = 1, lung metastasis: n = 1), where WB-DWI/MRI was correct. The 4 patients with operable distant metastases (< 3 liver metastases: *n* = 2, abdominal wall metastases: n = 2) were correctly assessed by WB-DWI/MRI. CT underestimated 2 of these patients, see example in Fig. 4. WB-DWI/MRI correctly identified all 12 patients with inoperable distant metastases, CT only 2.

#### Overall judgement of inoperability

WB-DWI/MRI was significantly better in the prediction of inoperability than CT (*P* < 0.00001), with a sensitivity of 90.6%, specificity 100%, PPV 100% and NPV 90.3% (no FP, 3 FN, 29 TP and 28 TN). For CT these values were 25.0, 92.9, 80.0 and 52.0%, respectively (2 FP, 24 FN, 8 TP and 26 TN). The 3 FN cases on WB-DWI/MRI were as follows: 1 patient with signet ring cell differentiation (known to have only little diffusion restriction) was able to undergo a complete CRS-HIPEC but developed recurrent disease within 3 months and was interpreted as a false negative result, 1 patient with multiple implants on the small bowel serosa, detected by WB-DWI/MRI, but with many chemotherapy effects (Fig. 1); the patient was given benefit of the doubt, and 1 patient with juvenile polyposis and mucinous differentiation, possibly suggestive of rapid disease progression.

## Discussion

Patients with colorectal peritoneal metastases benefit from accurate preoperative information on cancer spread, maximizing the chance of complete resection and improved survival. The present study showed that for peritoneal carcinomatosis from colorectal origin WB-DWI/MRI was highly accurate for the prediction of inoperability (PPV 100%, NPV 90.3%), capable of detecting metastases in- and outside the abdominal cavity at the same time. On WB-DWI/MRI, disease burden was not overestimated, thus no patients were incorrectly deprived from surgery. WB-DWI/MRI only underestimated disease extent in 3 patients (5%). Two of these patients had special subtypes of cancer (signet ring cell and mucinous adenocarcinoma), known for their limited diffusion restriction and more aggressive nature, and in one patient residual disease (in combination with therapy response) was seen on MRI but he was given the benefit of the doubt, because of his young age. Although accurate assessment of the WB-DWI/MRI is often possible, the radiologist should be aware that in some circumstances the interpretation of the MR images is more tempting. In contrast to the 5% underestimation in WB-DWI/MRI, CT underestimated disease extent in 24 patients (40%), potentially leading to unnecessary explorative laparotomies.

To the best of our knowledge, the present study is the largest WB-DWI/MRI study of colorectal PC patients specifically addressing the estimation of overall operability, which has a high clinical relevance. Several other studies involving tumours with different histopathology (mainly ovarian, colorectal and appendiceal) have evaluated the accuracy of CT, PET/CT and/or MRI for the detection of PC. CT had an insufficient performance to detect PC with accuracies around 51–88% and severe underestimation of the PCI [10, 19, 20], whereas MRI-based PCI had a good correlation with surgical PCI in our study and others [20, 21]. Moreover, CT had the most difficulties in detecting lesions < 1 cm [10, 22] and serosal carcinomatosis on the small bowel and its mesentery (region 9–12), with accuracies ranging of 21–48% [10, 19, 20, 23]. Extensive serosal involvement often is a cause of inoperability. Granting that image interpretation is sometimes challenging because of physiologically high signal intensity of the bowel structures (for example seen in Fig. 1), DWI/MRI is known to be very good at illustrating tumour at the small bowel wall with accuracies of 92–95% [20]. This is in line with the present findings. This study showed NPVs of 100% for small bowel serosal metastases, with important implications for clinical/surgical decision making. Two studies found better performance of PET/CT than CT, but results were not statistically significant [24, 25]. One study found equal accuracy for PET/CT and abdominal MRI for the detection of PC, whereas MRI was better at detection of metastases in the surgically critical supramesocolic area [26].

Although this is a large single centre study on consecutive patients with PC from colorectal origin, we acknowledge some limitations. A heterogeneous patient population was included, presenting both with primary and recurrent tumours, treated with or without chemotherapy. Nonetheless, this may show the strength of WB-DWI/MRI in different settings. Another limitation of the study may be that the WB-DWI/MRI results of one experienced radiologist were examined. However, in the study of Michielsen et al. [27] WB-DWI/MRI showed almost perfect interobserver agreement for prediction of incomplete resection in patients suspected for ovarian cancer. We accepted CT as imaging method for comparison with the WB-DWI/MRI as various studies showed comparable results of CT and PET/CT [24, 25].

Currently, MRI plays an important role in the assessment of colorectal cancer, mainly used for local staging of rectal cancer and detection of liver metastases [28]. With the results of this study we can conclude that WB-DWI/MRI is a potentially powerful imaging method that, with a single time-efficient examination, provides all important information about disease extent and disease location needed to decide whether a patient with colorectal PC can undergo successful CRS with HIPEC.

## Conclusions

In conclusion, WB-DWI/MRI significantly outperformed CT for estimation of spread of PC, overall staging and prediction of operability. Pending validation in larger prospective studies, WB-DWI/MRI could act as guidance for surgical planning, minimize unnecessary exploratory laparotomies and, by directing the surgeons to all the correct disease locations, maximize the number of complete resections.

## Abbreviations

^18^F-FDG-PET/CT: Fluorine-18 fluorodeoxyglucose positron emission tomography/computed tomography
CRS-HIPEC: Cytoreductive surgery - hyperthermic intraperitoneal chemotherapy
CT: Computed tomography
WB-DWI/MRI: Whole-body diffusion-weighted magnetic resonance imaging
